# Changes in sleep pattern and dream activity across and after the COVID‐19 lockdown in Italy: A longitudinal observational study

**DOI:** 10.1111/jsr.13500

**Published:** 2021-10-01

**Authors:** Valentina Alfonsi, Maurizio Gorgoni, Serena Scarpelli, Pierpaolo Zivi, Stefano Sdoia, Emanuela Mari, Alessandro Quaglieri, Fabio Ferlazzo, Anna Maria Giannini, Luigi De Gennaro

**Affiliations:** ^1^ Department of Psychology Sapienza University of Rome Rome Italy; ^2^ Body and Action Lab IRCCS Fondazione Santa Lucia Rome Italy

**Keywords:** COVID‐19 lockdown, dreaming, longitudinal study, sleep diary, sleep pattern

## Abstract

A large body of evidence has documented the impact of the global COVID‐19 outbreak – and especially the lockdown period – on sleep quality and quantity. Here, we present the first Italian longitudinal study on sleep and COVID‐19 considering four different time points collected during lockdown (from 29 March 2020 to 3 May 2020) and a subsequent follow‐up period (October 2020). We used an online survey to collect socio‐demographic and COVID‐19 related information. Subjects were also asked to complete a sleep diary at each time point of the study. Our longitudinal sample included 147 participants. Statistical comparison across time intervals showed remarkable changes in sleep patterns during and after the lockdown. In particular, during lockdown we observed longer sleep latency, less ease of falling asleep, a higher total bedtime, and a lower dream frequency. The week‐by‐week evaluation described relatively stable patterns in the observed measures during the lockdown period, except for dream frequency, affected by a rapid increase in the early phase of lockdown. Our findings are in line with the current literature. Furthermore, the prospective longitudinal investigation comprising several time points offered the possibility of (a) observing the temporal dynamics and the different entities of such changes over time, and (b) reducing the typical memory bias for these studies.

## 1 INTRODUCTION

The outbreak of the novel coronavirus disease 2019 (COVID‐19) globally impacted the population. Beyond the high risks directly arising from the infectious disease, the current pandemic represented an unprecedented change in lifestyle and daily routine.

In Italy – one of the first European countries affected by the infection – several restrictive measures were implemented to mitigate the rapid spread of COVID‐19. In the first phase of the pandemic, the Italian Government imposed a total lockdown (March 9 to May 4, 2020), forcing its inhabitants to social distancing and home confinement.

Not surprisingly, all these substantial changes led to an increase in psychological problems among the general population, as demonstrated by the high levels of anxiety, depression, and post‐traumatic stress symptoms (Torales et al., 2020).

Although the adverse effects of the COVID‐19 pandemic on mental health were well documented all over the world, the consequences on the sleep pattern described a complex scenario. In line with timely warnings stated by a task force of the European Academy of Cognitive‐Behavioral Treatment of Insomnia in the first months of 2020 (Altena et al., 2020), current findings showed both worsening and improving aspects of sleep following the adoption of the restrictive measures (Alfonsi et al., 2021).

In general, most studies described an increase in sleep‐related disturbances, defining a global phenomenon called “COVID‐somnia” (Gupta & Pandi‐Perumal, 2020). Insomnia complaints during pandemic were reported with 20–45% prevalence among the general population worldwide (Becker et al., 2021). However, the results were not uniform across different populations or specific sleep domains. On the one hand, evidence showed that people went to bed and woke up later during lockdown (Cellini et al., 2020; Gupta, 2020; Wright et al., 2020), along with a longer time spent in bed (Blume et al., 2020; Li et al., 2020; Marelli et al., 2021; Wright et al., 2020). On the other hand, the time effectively spent sleeping – and consequently the sleep efficiency – underwent a parallel decrease (Blume et al., 2020; Casagrande et al., 2020; Franceschini et al., 2020; Gupta, 2020), as possibly documented by the high level of hypnotic uptake (Beck et al., 2020) to compensate the sleep debt.

As expected, people directly facing the disease – such as healthcare workers or COVID‐19 patients – had experienced a higher risk of developing sleep problems and psychological distress (Jahrami et al., 2021). Moreover, sociodemographic factors such as female gender were associated with higher mental distress and poorer sleep quality (Salfi, Lauriola, et al., 2020).

Mental activity during sleep also underwent relevant quantitative and qualitative changes during the pandemic. Recent national surveys showed increased dream and nightmare recall frequency during the Italian lockdown (Gorgoni et al., 2021; Scarpelli, Alfonsi, et al., 2021). Moreover, waking distressing events caused by the spread of the virus affected the subsequent dream features, in line with the well‐known “continuity‐hypothesis” (Schredl, 2006). Namely, many studies described higher negative contents and emotional charge in dream reports compared with the pre‐pandemic period (Gorgoni et al., 2021; Iorio et al., 2020; MacKay & DeCicco, 2020; Mota et al., 2020).

Overall, the above‐mentioned findings point out that the pandemic – particularly the home confinement period – led to drastic and complex consequences on sleep and dreaming activity. However, it is worth noting that most studies on sleep and COVID‐19 adopted a cross‐sectional design (Alimoradi et al., 2021). Despite these studies having the benefit of rapidly collecting data from a large sample and on a broad range of variables – especially during the ongoing COVID‐19 era – they do not allow the establishment of any cause‐and‐effect relationship. A limited number of studies tried to explore more in‐depth the dynamics of the observed effects using longitudinal designs (Alfonsi et al., 2021; Beck et al., 2021; Saraswathi et al., 2020; Scarpelli, Gorgoni, et al., 2021; Zhang et al., 2020). Notably, most of the current longitudinal studies on sleep aspects during the pandemic referred to a single time point throughout lockdown, compared with a baseline (pre‐lockdown) or a follow‐up (post‐lockdown) period.

Here, we present the first, to our knowledge, Italian longitudinal study on sleep and COVID‐19 considering multiple time points collected during and after the lockdown period. In particular, we aimed to describe the temporal dynamics of sleep and dream‐related aspects collected by prospective diaries at different time points during the course of lockdown and in correspondence of a follow‐up period, when the measures were gradually eased. The main advantage of this study concerns the opportunity to observe not only the difference between conditions during and after (or before) the lockdown period but also the temporal evolution of sleep patterns during the different phases of lockdown, revealing the possible influence of the passage of time (i.e., adaptation effects).

Starting from these assumptions, the aims of the present study were (a) to confirm the significant changes in several sleep domains during and after lockdown, accordingly with previous literature, and (b) to explore for the first time the temporal evolution of these sleep‐related changes across different time points of lockdown.

## 2 METHODS

### 2.1 Participants and procedure

This longitudinal study started in March 2020 (during the fourth week of the national lockdown) and concluded in October 2020 (four months after the end of the national lockdown). Participants were recruited through online advertisements on websites and social media platforms. The inclusion criteria were: age at least 18 years old and having electronic devices and an internet connection available to complete the questionnaire.

Baseline measures (demographic, COVID‐19‐related, and sleep quality features) were collected at the beginning of the study using a web‐based survey implemented on the Qualtrics Survey Platform and shared via email.

Longitudinal data (online sleep diary) were collected at weekly intervals during the fourth (March 29 to April 5, T1), the fifth (April 6 to April 12, T2), the sixth (April 13 to April 19, T3) and the eighth/last (April 27 to May 3, T4) week of lockdown and during a final follow‐up week in the post‐lockdown period (September 14 to October 12, Follow‐up, FU). Therefore, we had three consecutive time intervals during lockdown (T1, T2, T3), one interval as the last week of lockdown (T4), and one post‐lockdown measurement (FU).

All individuals explicitly agreed to participate in the research and completed the survey after reading and signing the electronic informed consent. Participants could withdraw from the study at any moment, and identification codes were created to anonymize personal data.

The study was conducted in accordance with the Declaration of Helsinki and approved by the Institutional Review Board of the Department of Psychology of the Sapienza University of Rome (Prot. #577, March 28, 2020).

Data reported in this study were part of a wider longitudinal research project concerning the psychological impact of home confinement in Italy. Other data with different purposes have been presented elsewhere (Alfonsi et al., 2021; Quaglieri et al., 2021).

### 2.2 Measures

#### 2.2.1 Web‐based survey

*Socio*‐*demographic and COVID*‐*19 related information*: an initial questionnaire was administered to assess socio‐demographic characteristics, such as gender, age, educational level, occupation, marital status. A subsequent set of questions assessed COVID‐19 related information (e.g., COVID‐19‐infected relatives or friends, forced quarantine period).

*Sleep quality measures*: Sleep quality was assessed by the Pittsburgh Sleep Quality Index (PSQI) (Curcio et al., 2013). The PSQI is a well‐known self‐report questionnaire investigating sleep quality over one month. It comprises 19 items, from which partial scores in seven subscales (ranging from 0 to 3) and a global score (ranging from 0 to 21) are calculated. The subscales refer to seven different components: subjective sleep quality, sleep latency, sleep duration, sleep efficiency, sleep disturbances, use of sleep medications, daytime dysfunction. A PSQI global score >5 indicates poor sleep quality.

#### 2.2.2 Sleep diary

An online‐adapted version of a sleep diary was used. Participants were asked to fill out the sleep diary within 30 minutes after the final morning awakening. It collected information on several sleep‐related variables, such as sleep onset latency (SOL, min: the amount of time it takes to fall asleep after the lights have been turned off), ease of falling asleep (the subjective estimation of the ability to fall asleep on 6‐point Likert scale: from very difficult (1) to very easy (6)), number of awakenings across the night (NOA), subjective total sleep time (sTST, min: the subjective estimation of the amount of time spent sleeping), number of recalled dreams. We then extracted other information from the raw data of the sleep diary, such as total bed time (TBT, min: the amount of time from light off to rise time), total sleep time (TST, min: the amount of time spent sleeping), and sleep efficiency (SE, %: TST/TBT ×100).

### 2.3 Data analysis

Descriptive statistics were carried out for sociodemographic, COVID‐19 related and sleep related characteristics of the sample.

The sleep measures obtained from sleep diaries were individually averaged at each time‐point (T1, T2, T3, T4, and FU). The dependent variables were: (1) SOL, min; (2) ease of falling asleep; (3) NOA; (4) TBT, min; (5) TST, min; (6) SE, %; and (7) number of recalled dreams.

Statistical analyses were performed on longitudinal data by a one‐way repeated‐measures multivariate analysis of variance (MANOVA), with “Time” (T1, T2, T3, T4, FU) as within‐subjects factor and sleep measures as dependent variables. We then carried out univariate one‐way repeated‐measures ANOVAs as follow‐up analyses. Post hoc comparisons were conducted using the Least Significant Difference (LSD) method. Partial‐eta squared (η_p_^2^) has been used as a measure of effect size.

The normal distribution of the original data was checked and skewed data were transformed into normal distribution using square roots transformation (positive skew) and power transformation (negative skew).

All data were analyzed using Statistical Package for Social Science (SPSS; version 25.0; IBM SPSS, Armonk, NY) and Matlab R2019. Values of *P* ≤0.05 were considered statistically significant.

## 3 RESULTS

### 3.1 Sample characteristics

A total of 646 subjects took part in the study. We excluded 38 participants who completed the questionnaire more than once and 16 non‐Italian subjects. 445 subjects who retired or who did not participate in all the five time intervals were excluded according to the within nature of the longitudinal design. Our final longitudinal sample consisted of 147 subjects (mean age ± SD: 34.07 ± 15.89, age range: 18–81, 105 females). Demographic, COVID‐related, and initial sleep quality features are shown in Table 1.

**TABLE 1:** Descriptive characteristics of the longitudinal sample (*N* = 147)

	*N* (tot. 147)	%
*Demographic features*		
Gender		
Male	42	29%
Female	105	71%
Age		
18–30 years old	89	61%
31–50 years old	22	15%
>50 years old	36	24%
Education		
Until middle School	13	9%
High School	66	45%
Graduated	68	46%
Occupation		
Student	74	50%
Employed	60	41%
Unemployed or retired	13	9%
Marital status		
Single	102	70%
Married	39	26%
Separated/Divorced	6	4%
*COVID‐19‐related features*		
Infected relative/friend		
Yes	11	7%
No	136	93%
Forced quarantine		
Yes	5	3%
No	142	97%
*Sleep features*		
Self‐reported sleep quality		
PSQI > 5	69	47%
PSQI ≤ 5	78	53%

### 3.2 Longitudinal changes in sleep pattern across and after the lockdown period

**FIGURE 1:**
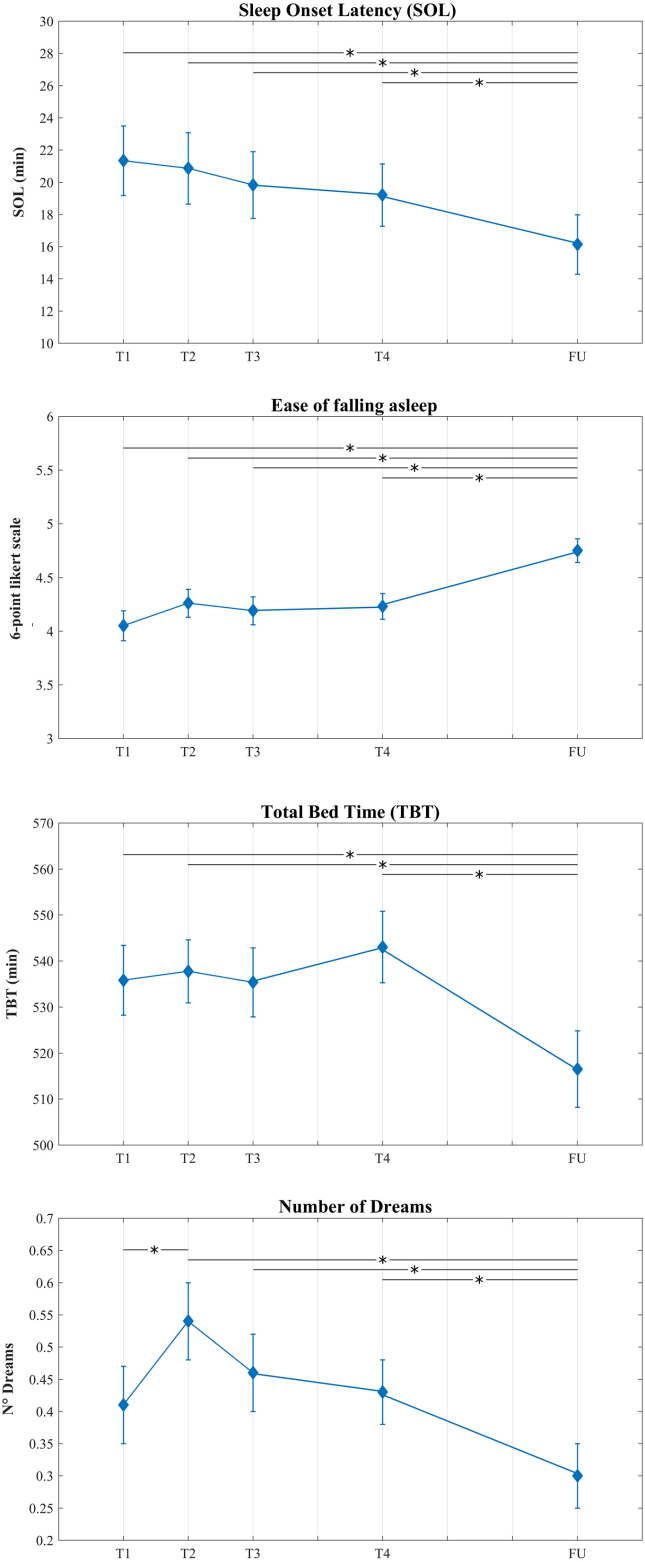
Means (and *SE*) across the time intervals (T1, T2, T3, T4, FU) for the following variables: Sleep onset latency (SOL), Ease of falling asleep, Total bed time (TBT), Number of dreams. *Significant at *P* ≤ 0.05 [Colour figure can be viewed at wileyonlinelibrary.com]

One‐way MANOVA comparing the sleep patterns across the different lockdown periods and a subsequent FU showed a statistically significant effect of “Time” (Wilks’*λ* = 0.654, *F*_28,119_ = 2.244, *P* = 0.001). The univariate repeated measures ANOVAs (Table 2) showed a significant main effect of “Time” for the following variables: sleep latency (*F*_4,584_ = 2.543, *P* = 0.042), ease of falling asleep (*F*_4,584_ = 6.892, *P* < 0.001), TBT (*F*_4,584_ = 2.731, *P* = 0.034) and number of recalled dreams (*F*_4,584_ = 4.224, *P* = 0.002). Results from significant LSD post hoc comparisons are reported in Figure 1. Namely, LSD post hoc comparisons revealed a significant reduction of SOL during the follow‐up period compared with all previous lockdown phases (T1 vs. FU: *P* = 0.004; T2 vs. FU: *P* = 0.017; T3 vs. FU: *P* = 0.041; T4 vs. FU: *P* = 0.022). In a specular way, the ease of falling asleep exhibited significantly higher scores during the post‐lockdown compared with all the lockdown time points (T1 vs. FU: *P* < 0.001; T2 vs. FU: *P* = 0.001; T3 vs. FU: *P* < 0.001; T4 vs. FU: *P* < 0.001).

**TABLE 2:** Univariate ANOVAs comparing the time intervals (T1 vs. T2 vs. T3 vs. T4 vs. FU). Bold values denote statistical significance at *P* ≤ 0.05

	Mean (*SE*)	*F*‐values	*P* values	*η_p_* ^2^
SOL (min)				
T1	21.33 (2.16)	**2.543**	**0.042**	**0.017**
T2	20.86 (2.22)			
T3	19.83 (2.07)			
T4	19.20 (1.93)			
FU	16.13 (1.85)			
Ease of falling asleep				
T1	4.05 (0.14)	**6.892**	**<0.001**	**0.045**
T2	4.26 (0.13)			
T3	4.19 (0.13)			
T4	4.23 (0.12)			
FU	4.75 (0.11)			
NOA				
T1	0.99 (0.11)	1.753	0.137	0.012
T2	0.90 (0.10)			
T3	1.11 (0.11)			
T4	1.14 (0.12)			
FU	0.95 (0.12)			
TBT (min)				
T1	535.81 (7.59)	**2.731**	**0.034**	**0.018**
T2	537.76 (6.88)			
T3	535.35 (7.50)			
T4	543.03 (7.77)			
FU	516.50 (8.32)			
TST (min)				
T1	462.89 (7.47)	0.891	0.469	0.006
T2	466.45 (6.95)			
T3	466.62 (6.88)			
T4	473.15 (7.02)			
FU	458.39 (7.51)			
SE (%)				
T1	86.86 (1.05)	1.644	0.162	0.011
T2	87.27 (0.99)			
T3	87.90 (0.94)			
T4	87.69 (0.87)			
FU	89.27 (0.83)			
Number of dreams				
T1	0.41 (0.06)	**4.224**	**0.002**	**0.028**
T2	0.54 (0.06)			
T3	0.46 (0.06)			
T4	0.43 (0.05)			
FU	0.30 (0.05)			

Concerning TBT, post hoc analyses revealed a significant reduction of time spent in bed during the follow‐up interval compared with almost all the lockdown time points (T1 vs. FU: *P* = 0.036; T2 vs. FU: *P* = 0.026; T4 vs. FU: *P* = 0.006).

Finally, regarding the number of dreams, we observed an initial increase in the second time interval compared with the first one (T1 vs. T2: *P* = 0.021) followed by a significant decrease during the follow‐up period (T2 vs. FU: *P* < 0.001; T3 vs. FU: *P* = 0.025; T4 vs. FU: *P* = 0.028).

## 4 DISCUSSION

In agreement with our hypotheses, we found significant changes in sleep‐related aspects during and after lockdown. In particular, we observed longer sleep latency and reduced ease of falling asleep during lockdown than post‐lockdown. In parallel, time spent in bed underwent a significant decrease during the follow‐up period compared with most of the intervals within the lockdown period. Furthermore, the longitudinal assessment including four consecutive lockdown intervals showed stable patterns of sleep measures (SOL, ease of falling asleep, TBT) regardless of the time passage, except for dream activity. Indeed, dream frequency exhibited an initial rapid increase in the early phase of lockdown and then constant higher scores compared with follow‐up.

In particular, the amount of time before falling asleep remained relatively stable across the lockdown period, with a significant reduction during the follow‐up phase compared with all lockdown intervals. A specular trend was found about the ease of falling asleep. We observed a significant peak of increase in the perceived ability to fall asleep during the post‐lockdown in contrast to the lower scores during the home confinement period. Both of these findings are expressions of the same phenomenon, and they are in line with previous results addressing longer sleep latency and reduced ease of falling asleep during the lockdown period compared with the return to usual schedules and daily routines (Alfonsi et al., 2021; Scarpelli, Gorgoni, et al., 2021).

Several hypotheses have been proposed to explain these changes, as a function of specific lockdown‐related aspects. For example, the increased electronic device usage near bedtime during the isolation period (Cellini et al., 2020; Salfi, Amicucci, et al., 2020) could exacerbate the subjective difficulty in falling asleep. Indeed, it is well established that the evening exposure to screen light may have detrimental effects on human sleep and circadian rhythms due to the lengthening of sleep latency and the increasing of intra‐sleep wakefulness (Green et al., 2017). Also, it is conceivable that the higher levels of stress and anxiety experienced during the social restriction period (for a review, see (Xiong et al., 2020)) may have caused an increase in physiological arousal. Consequently, longer sleep onset timing could reflect the known relationship between mental distress experienced during the day and the quality of subsequent sleep (Kalmbach et al., 2015). Furthermore, our sample consists mostly of women, who seem to be particularly vulnerable to the psychological consequences of the pandemic (Salfi, Lauriola, et al., 2020). In addition to mirroring the data on sleep latency, the perceived difficulty of falling asleep during lockdown can also be associated with the possible reduction of the homeostatic sleep pressure in that period. Indeed, the lack of traditional social, school, and work schedules caused remarkable changes in sleep timing and circadian rhythms (Blume et al., 2020).

Even the increase in TBT during lockdown could be explained within this theoretical framework. It should be hypothesized that people have adapted daily activities to their individual chronotype, and this allowed the re‐adjustment of the natural sleep‐wake schedule in the absence of social time cues (Leone et al., 2020). In general, studies worldwide described a shift to later bedtime and rise time during the quarantine period (Cellini et al., 2020; Gupta, 2020; Wright et al., 2020). Also, the reduced sunlight exposure may have concurred to the reduction of the externally imposed sleep‐wake rhythms (Smit et al., 2021). However, we should consider that the increase in time spent in bed was not paralleled by the same rise of the effective sleeping time. As a result, the global sleep efficiency was reduced compared with the pre‐pandemic period (Blume et al., 2020; Casagrande et al., 2020; Franceschini et al., 2020; Gupta, 2020).

The pattern of our results is consistent with previous literature (for a review, see Jahrami et al., 2021). Moreover, the divergent findings as a function of the specific sleep domain suggested the low ability of composite measures (e.g., PSQI) to provide an accurate picture of the multiple consequences of this unprecedented situation (Alfonsi et al., 2021).

Along with the above‐mentioned results concerning sleep, we also found significant changes across time points regarding the oneiric activity. In particular, we described a rapid increase in the number of dreams during the early phases of lockdown, reaching the highest peak in the second time interval and then gradually decreasing until the last week of lockdown. During the follow‐up period, we observed a drastic fall in the dream frequency compared with the confinement period. Consistently with previous literature, the increased dream rate during lockdown (Gorgoni et al., 2021; Iorio et al., 2020; Scarpelli, Alfonsi, et al., 2021; Schredl & Bulkeley, 2020) could be explained by a sort of “coping strategy” to metabolize stressful events and negative affect experienced during daytime life (Scarpelli et al., 2019). These results are also in keeping with previous studies showing significant changes in dream occurrence after experiencing traumatic events (e.g., Tempesta et al., 2013). Concerning the increased dream frequency in the early phase of lockdown, a possible explanation could be related to the “alarm reaction” phase occurring at the beginning of lockdown (higher levels of anxiety), followed by an adaptation to circumstances (Fancourt et al., 2021; Quaglieri et al., 2021). In line with the previously stated role of dreams during traumatic or stressful events, such a rapid increase may reflect the urgent need to adopt coping mechanisms at the very beginning of the overwhelming experience.

Furthermore, in previous research, higher dream frequency was also related to lower sleep quality – specifically to sleep fragmentation and intra‐sleep wakefulness (Schredl & Reinhard, 2008; Wyk et al., 2019). In particular, we could speculate that the changes in sleep timing during the lockdown (e.g., the later bedtime and rise time, the longer TBT) may have increased both the probability of REM awakenings and the number of REM‐wake transitions and – consequently – the recall of dreams upon morning awakenings (Koulack & Goodenough, 1976; Scarpelli et al., 2020). However, we could not directly establish this relationship given the lack of corresponding measures in the current study.

To the best of our knowledge, this is the first longitudinal study on sleep and dreams, collecting prospective data through repeated measures during an extended period (from March to October 2020) of the ongoing COVID‐19 pandemic. The within‐subjects design allowed us to observe the subjective trajectories of sleeping and dream activity during the different phases of lockdown and post‐lockdown. This aspect represents a great advantage compared with cross‐sectional studies, which provide a single snapshot of a given moment in time, without any information about what occurs immediately before and after that specific period.

Despite our study focused on the first lockdown period (“first contagion wave” – spring 2020), it is worth mentioning other national studies also considering the second lockdown period (“second contagion wave” – autumn 2020), characterized by lower sleep restrictions (Conte et al., 2021; Salfi et al., 2021). Interestingly, their results showed the high and permanent sensibility of sleep and dreaming to lifestyle modification, as reflected by the changes in both sleep quality (Salfi et al., 2021) and dream activity (Conte et al., 2021) closely related to the course of the pandemic. Overall, this evidence confirms the current results and allows us to read them from a wider time perspective.

Some methodological constraints of the present study should be taken into account. Firstly, the absence of any pre‐pandemic information prevented understanding the role of preexistent sleep and clinical features in affecting the nature of pandemic‐related changes. Another intrinsic limitation concerns the lack of objective sleep measures, which could have boosted what we observed from a subjective perspective. Furthermore, the online recruitment strategy may have introduced a self‐selection bias inherent to the web‐based studies. Anyway, we should consider that the longitudinal collection of prospective data potentially represents the best solution to describe the actual scenario referred to the selected variables, although it could further imply issues of self‐selection. Indeed, the retrospective assessment used in most studies allowed a rapid data collection from a large sample but affected the reliability of results due to the underlying memory bias (under‐ or overestimate).

Regarding the investigation about dream activity, we did not collect dream reports. Consequently, we only described the quantitative aspect of dreams without any reference to the nature of the contents and their possible relation with waking experiences.

Finally, our sample is not fully representative of the Italian population due to the unbalanced dataset (e.g., gender, age, occupation, marital status), and so caution is needed in interpreting and generalizing the results.

## 5 CONCLUSIONS

Our findings were consistent with previous literature showing lockdown as a key factor responsible for the reported changes in sleep and dream activity during the pandemic. Furthermore, the longitudinal investigation comprising several time points offered the possibility to observe the temporal dynamics and the different entities of these changes over time.

To sum up, our pattern of results pointed out two main conclusions (a) sleep‐related aspects underwent critical changes during lockdown: reduced ability to fall asleep (timing and ease), increased amount of time spent in bed, and higher dream recall frequency; (b) sleep pattern observed during lockdown remained quite stable over time, except for dream activity: an initial rapid increase was observed, followed by a partial stabilization.

The understanding of the effects of the pandemic on sleeping and dreaming is still under investigation. Further longitudinal studies should be conducted to shed more light on the evolution of such effects over a period of time and in different phases of the pandemic.

## ACKNOWLEDGEMENTS

Open Access Funding provided by Universita degli Studi di Roma La Sapienza within the CRUI‐CARE Agreement. [Correction added on 4 June 2022, after first online publication: CRUI funding statement has been added.]

## CONFLICT OF INTEREST

None of the authors have potential conflicts of interest to be disclosed.

## AUTHOR CONTRIBUTIONS

VA, MG, FF, AMG and LDG conceived and designed the experiment; SSc, PZ, SSd, EM, AQ collected and pre‐processed the data; VA and MG analyzed the data; VA, MG and LDG wrote the paper.

## Data Availability

The data that support the findings of this study are available from the corresponding author upon reasonable request.
